# Injection-modulated polarity conversion by charge carrier density control via a self-assembled monolayer for all-solution-processed organic field-effect transistors

**DOI:** 10.1038/srep46365

**Published:** 2017-04-12

**Authors:** Jeongkyun Roh, Taesoo Lee, Chan-mo Kang, Jeonghun Kwak, Philippe Lang, Gilles Horowitz, Hyeok Kim, Changhee Lee

**Affiliations:** 1 Department of Electrical and Computer Engineering, Inter-University Semiconductor Research Center, Seoul National University, 1 Gwanak-ro, Gwanak-gu, Seoul 08826, Korea; 2 IT Convergence Technology Research Laboratory, Electronics and Telecommunications Research Institute, Deajeon, Korea; 3 Department of Electrical and Computer Engineering, University of Seoul, 163 Seoulsiripdaero, Dongdaemun-gu, Seoul 02504, Korea; 4 ITODYS, CNRS UMR 7086, Université Paris Diderot (Paris7), 15 rue Jean-Antoine de Baïf, 75205 Paris Cedex 13, France; 5 LPICM, Ecole Polytechnique, CNRS, 91128 Palaiseau, France; 6 Construction Equipment Technology Center, Korea Institute of Industrial Technology (KITECH), Hayang-ro 13-13, Gyeongsan 38430, Korea

## Abstract

We demonstrated modulation of charge carrier densities in all-solution-processed organic field-effect transistors (OFETs) by modifying the injection properties with self-assembled monolayers (SAMs). The all-solution-processed OFETs based on an n-type polymer with inkjet-printed Ag electrodes were fabricated as a test platform, and the injection properties were modified by the SAMs. Two types of SAMs with different dipole direction, thiophenol (TP) and pentafluorobenzene thiol (PFBT) were employed, modifying the work function of the inkjet-printed Ag (4.9 eV) to 4.66 eV and 5.24 eV with TP and PFBT treatments, respectively. The charge carrier densities were controlled by the SAM treatment in both dominant and non-dominant carrier-channel regimes. This work demonstrates that control of the charge carrier densities can be efficiently achieved by modifying the injection property with SAM treatment; thus, this approach can achieve polarity conversion of the OFETs.

In the last decade, the field of organic electronics has seen dramatic progress in device physics and applications such as organic light-emitting diodes (OLEDs), organic photovoltaics (OPVs), and organic field-effect transistors (OFETs). Among these, OFETs have garnered tremendous attention because of their potential use in low-cost, lightweight and flexible electronic devices^1^,^2^,^3^,^4^,^5^,^6^.

One of the special features of organic semiconductors that distinguish these materials from other types of semiconductors such as silicon and amorphous metal oxides is their intrinsic ambipolar behaviour: organic semiconductors can transport both holes and electrons^7^,^8^. Ambipolar characteristics of organic semiconductors are expected to lead to a further reduction of processing costs because complementary metal oxide semiconductor (CMOS)-like integrated circuits can be fabricated with a single material, implying less deposition and patterning processing^9^,^10^.

In most cases, OFETs are classified as either p-type or n-type depending on the dominant charge carriers of the organic semiconductors. Because of their unbalanced hole and electron densities, unipolar OFETs are more common than ambipolar ones because the density of the dominant carriers overwhelms the density of the non-dominant carriers. However, when the density of the non-dominant carriers increases and becomes non-negligible, the ambipolar characteristics of the OFETs begin to appear. Furthermore, if the density of the non-dominant carriers surpasses the density of the dominant carriers, the OFETs return to the unipolar state but with reversed polarity. This phenomenon is referred to as ‘polarity conversion’ and has been observed in several OFET systems^11^,^12^,^13^. That is, the types of OFETs (i.e., unipolar or ambipolar) and the polarity of OFETs (i.e., p-type or n-type) are not determined by the intrinsic nature of the organic semiconductors themselves but rather by the densities of the dominant and non-dominant carriers. Exploiting this unique property of polarity conversion, researchers can fabricate organic CMOS-like integrated circuits using a single organic semiconductor, which implies a further reduction of the processing cost. Because polarity conversion can be enabled by controlling the densities of dominant and non-dominant carriers, modulation of charge carrier densities is important for achieving low-cost electronics using OFETs.

Previously, control of the charge carrier densities in OFETs has been accomplished by various methods. Molecular doping, where additional charge carriers are generated from the employed dopant materials, is one of the easiest ways to change the charge carrier densities^14^,^15^,^16^,^17^. Interface engineering at the organic semiconductor–gate insulator is another efficient method for controlling the charge carrier densities. Accumulated charges form a conductive channel in the OFETs near the organic semiconductor-gate insulator interface, and the properties of the interface substantially influence charge accumulation^18^,^19^,^20^,^21^. Lastly, Schottky barrier control has been often used to adjust charge carrier densities. The injected carrier density can be modulated through tuning of the injection property^22^,^23^,^24^.

Among these methods, we focused on controlling the charge carrier densities by modification of the injection property in OFETs because of two particular advantages: compatibility with a wide range of systems and suitability for studies of the intrinsic nature of charge transport in organic semiconductors. Whereas the initially described method of molecular doping enables easy tuning of the charge carrier densities, doping conditions (i.e., dopant identity and doping ratio) should be optimized carefully depending on each system. Furthermore, molecular doping is not suitable for studying the intrinsic properties of organic semiconductors because the bulk properties are substantially changed by the employed dopant. The second described method, interface engineering at the organic semiconductor–gate insulator interface, also exhibits a drawback in terms of universality. In this method, the charge carrier densities are controlled via an induced dipole from the interfacial layer between, for example, a self-assembled monolayer (SAM) and a high-permittivity polymer. However, introducing an interfacial layer between the organic semiconductor and gate dielectric has many limitations because it can be only applied to certain interfaces. By contrast, injection barrier control for modulating charge carrier densities has many advantages in that it is compatible with various systems and is also suitable for studying the intrinsic charge transport characteristics of the organic semiconductor.

In this study, we used SAMs to demonstrate injection-modulated charge carrier densities in all-solution-processed OFETs. Solution-phase processability is one of the most appealing aspects of OFETs for achieving low-cost electronics; we therefore employed all-solution-processed OFETs as a test platform in this study. We implemented the all-solution-processed OFETs based on an n-type polymer with inkjet-printed Ag electrodes and then modulated electron densities by modifying the injection property of the OFETs. The inkjet-printed Ag electrodes were modified by two types of SAMs with different dipole directions: thiophenol (TP) and pentafluorobenzene thiol (PFBT). The modification of the injection property by the SAMs was investigated using ultraviolet photoelectron spectroscopy (UPS). As a result of the SAM treatment, electron density was controlled according to the injection barrier height. The electron density was investigated using the parallel capacitance model, and the relevant OFET parameters such as the electron field-effect mobility, turn-on voltage, and contact resistance of the OFETs were investigated according to the SAM treatment. Furthermore, we also investigated the density modulation of the non-dominant carriers (i.e., holes) by employing the SAMs in the all-solution-processed n-type OFET.

## Results

**Figure 1:**
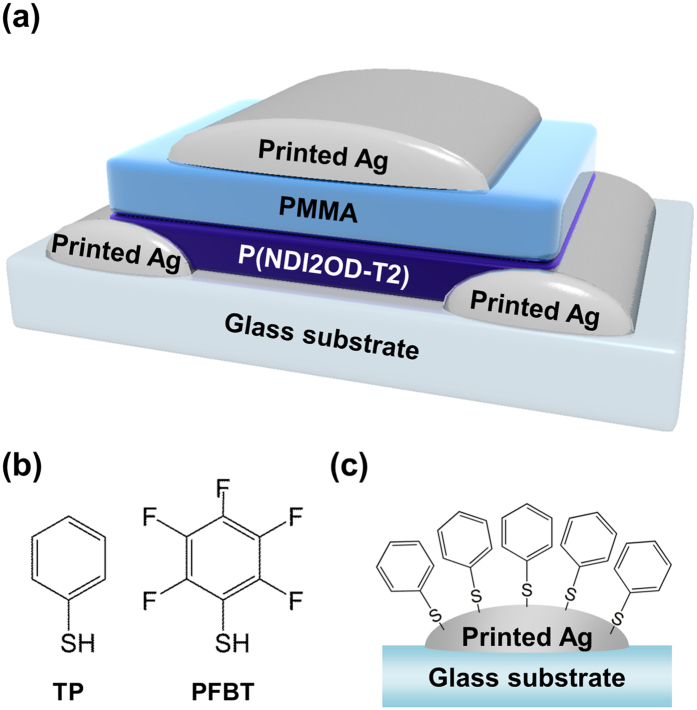
Illustration of OFETs and chemical structure of SAMs. (**a**) Device structure of the all-solution-processed OFETs. (**b**) Chemical structure of the SAMs and (**c**) illustration of the inkjet-printed Ag electrode with TP treatment.

As shown in Fig. 1(a), the all-solution processed OFETs with a top-gate bottom-contact configuration were fabricated. TP and PFBT were used to modify the inkjet-printed Ag electrodes; their chemical structures are shown in Fig. 1(b). Figure 1(c) illustrates an example of inkjet-printed Ag electrodes with SAM treatment.

**Figure 2:**
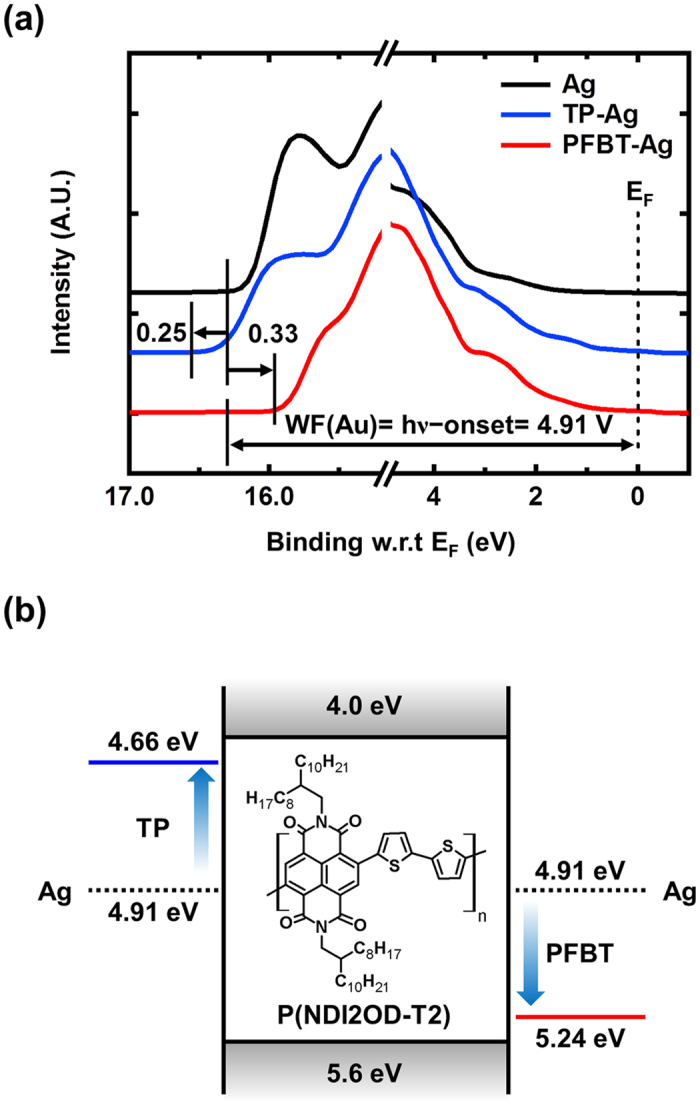
UPS spectra of SAM-treated Ag and energy diagram at the contact region (**a**) UPS spectra of the inkjet-printed Ag electrodes with and without SAM treatment. (**b**) Energy diagrams of the charge injection at the interface between P(NDI2OD-T2) and inkjet-printed Ag electrodes with and without SAM treatment.

Modulation of the charge carrier densities in the all-solution-processed OFETs was facilitated by modifying the injection properties. Because the injection barrier is determined by the Schottky barrier height between the electrodes and organic semiconductor, we used SAMs to modify the injection properties. The inkjet-printed Ag electrodes were treated with the two types of SAMs with opposite dipole directions, TP and PFBT. We first measured the modified work function of the SAM-treated Ag electrodes using UPS. Figure 2(a) shows the normalized UPS spectra of the inkjet-printed Ag electrodes with and without the SAM treatment. Increased binding energy of the secondary cut-off edge implies a reduced work function of the inkjet-printed Ag electrodes because of the TP treatment, whereas the decreased binding energy of the secondary cut-off edge indicates an increased work function of the inkjet-printed Ag electrodes because of the PFBT treatment. On the basis of the UPS results, the work function of the inkjet-printed Ag was determined to be 4.91 eV and was determined to change to 4.66 and 5.24 eV after the TP and PFBT treatments, respectively. Figure 2(b) shows the energy diagram of the charge injection at the interface between the metal and organic semiconductor. As described in Fig. 2(b), the lowest unoccupied molecular orbital (LUMO) is located on the introduced n-type polymer, P(NDI2OD-T2) and the highest occupied molecular orbital (HOMO) is located on the P(NDI2OD-T2), with energies of 4.0 eV and 5.6 eV, respectively^25^. The electron injection barrier can be estimated by the Mott-Schottky model that defines the injection barrier as the difference between the work function of metals and the LUMO level of P(NDI2OD-T2). In the absence of the SAM treatment, a high injection barrier of 0.91 eV from the electrode to the n-type polymer was obtained. After the TP treatment, the work function of the inkjet-printed Ag electrodes was modified to 4.66 eV, resulting in a lower electron injection barrier of 0.66 eV with P(NDI2OD-T2). Conversely, PFBT treatment led to the increased work function of the inkjet-printed Ag electrodes because of the opposite dipole direction, yielding a further increase of the electron injection barrier to 1.24 eV. As observed by the UPS measurement, the electron injection barrier from the electrode to the organic semiconductor was modulated by SAM treatment.

**Figure 3:**
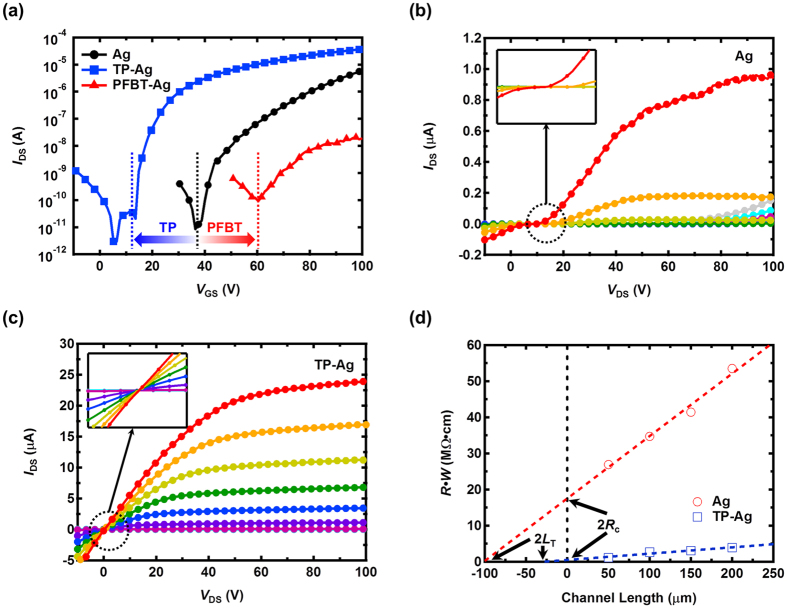
Transfer and output characteristics of the n-channel OFETs and contact resistance extraction. (**a**) Transfer characteristics of the all-solution-processed OFETs with and without SAM treatment. Output characteristic of the device (**b**) without SAM treatment and (**c**) with TP treatment. Output characteristics were obtained in the region of gate-to-source voltage from 0 V to 80 V with a 10 V step. (**d**) Width-normalized device resistance with respect to the channel lengths.

As a next step, we investigated the effect of the modulated injection barrier on the electrical performance of the all-solution-processed OFETs. Figure 3(a) shows the transfer characteristics of the all-solution-processed n-channel OFETs realized by the inkjet-printed Ag electrodes with and without SAM treatment. The transfer characteristics were obtained in the saturation regime where *V*_GS_ = 70 V. As evident in Fig. 3(a), a dramatic change in the transfer characteristics was observed after the SAM treatment. We first examine the substantial shifts of the turn-on voltage (*V*_ON_) induced by the SAM treatment. The turn-on voltage is the gate-to-source voltage at which the accumulated charges start to induce a conducting channel; this voltage is thus strongly related to the charge carrier densities. A lower turn-on voltage implies easier induction of the conducting channel because of the higher charge carrier density. Prior to the SAM treatment, the device exhibited a high turn-on voltage of 38.8 V, which is attributed to the considerable injection barrier from the inkjet-printed Ag electrodes. After the TP treatment, the turn-on voltage shifted in a negative direction to 13.6 V as a result of the reduced electron injection barrier. By contrast, the PFBT treatment yielded a shift in the turn-on voltage in a positive direction to 60.4 V, representing suppressed electron injection. The field-effect mobility (*μ*_FET_) and the threshold voltage (*V*_TH_) can be extracted from the equation


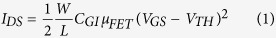


where *I*_DS_ is the drain-to-source current, *C*_GI_ is the capacitance of the gate insulator per unit area, and *V*_GS_ is the gate-to-source voltage. The extracted electron field-effect mobilities of the devices without the SAM treatment and with TP and PFBT treatment were 0.021 cm^2^/V·s, 0.11 cm^2^/V·s, and 2.4 × 10^−4^ cm^2^/V·s, respectively. Because the charge carrier densities modulated by the injection property determine the channel conductance in the devices, a dramatic variation of the electron field-effect mobility was observed. Compared to the electron mobility of the device without the SAM treatment, the device with the TP treatment exhibited a greater than fivefold increase in electron mobility, whereas the electron mobility of the device with the PFBT treatment was reduced by two orders of magnitude. In the case of the threshold voltage, a trend similar to that of the turn-on voltage was observed. The threshold voltages of the devices without the SAM treatment, with the TP treatment, and with the PFBT treatment were 53.7 V, 17.3 V, and 60.2 V, respectively. All parameters obtained in the n-channel OFETs are summarized in Table 1.

On the basis of the threshold voltage difference of the devices, we estimated the additional electron density (Δ*n*_e_) accumulated by the SAM treatment using the parallel capacitance model. This model leads to derivation of the relationship Δ*n*_e_ = *C*_GI_Δ*V*_TH_/*e*, where Δ*V*_TH_ is the threshold voltage difference of the devices and *e* is the elementary charge (*e* = 1.602 × 10^−19^ C). The additional modulated electron density obtained by the lowering of the Schottky barrier with the TP treatment was calculated to be 6.82 × 10^11^ cm^−2^. In the OFET with the PFBT treatment, the electron density was reduced by 1.21 × 10^11^ cm^−2^ because of the increased Schottky barrier.

Figure 3(b,c) show the output characteristics of the devices without the SAM treatment and with the TP treatment, respectively. The device without the SAM treatment do not exhibit good saturating behaviours in the low gate-to-source voltage regime (*V*_GS_ < 70 V) because of the low electron density. Furthermore, diode-like current-voltage behaviours were observed in the low gate-to-source voltage regime, which indicates a contribution of non-dominant carrier (hole) transport. The non-Ohmic behaviour in the early part of the output characteristics (i.e., low drain-to-source voltage regime) implies that electron injection is substantially limited by the Schottky barrier. By contrast, the device with TP treatment exhibits good saturating behaviours under the low gate-to-source voltage regime as a result of increased electron density. The Ohmic behaviour in the output characteristic indicates the efficient electron injection from the electrodes.

For a more quantitative investigation of the contact properties of the devices without SAM treatment and with TP treatment, the transmission line method (TLM) was employed^26^,^27^. Figure 3(d) shows the plot of the width-normalized device resistance (*R*·*W*) with respect to the channel lengths (50 μm, 100 μm, 150 μm, and 200 μm). The device resistances were calculated from the slope of the output curves at the gate-to-source voltage of 80 V, and the contact resistances were obtained from the y-intercept of the graphs. The extracted width-normalized contact resistance (*R*_c_·*W*) of the device without SAM treatment and with TP treatment are 1.75 × 10^7^ Ω·cm and 4.80 × 10^5^ Ω cm, respectively. The transfer length (*L*_T_) is the effective length of the electrodes that actually participate in the charge injection^28^,^29^, and the extracted transfer lengths are 50.6 μm and 13.7 μm for the devices without the SAM treatment and with the TP treatment, respectively. The transfer length of the device with the TP treatment is comparable to the value based on vacuum-deposited electrodes with a top-contact geometry reported in previous studies, implying a good contact property of the TP-treated Ag electrodes with the n-type organic semiconductors^29^.

**Figure 4:**
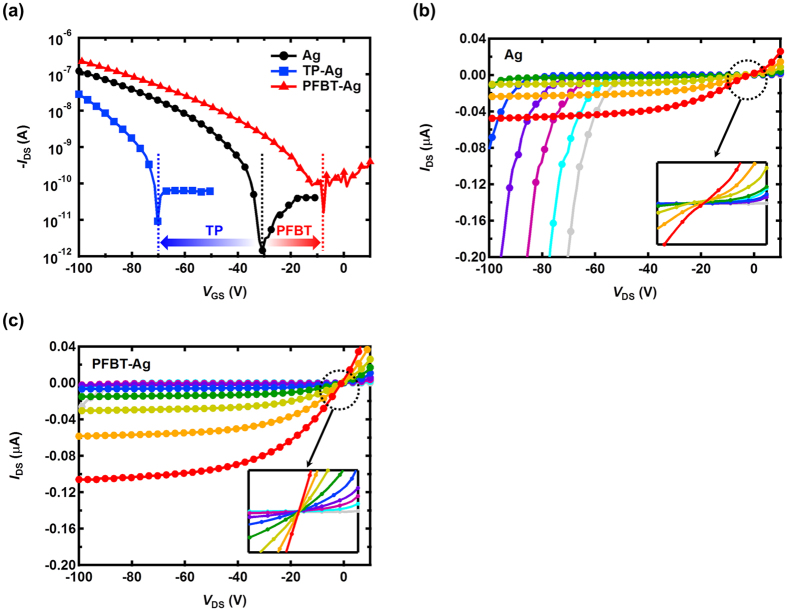
Transfer and output characteristics of the p-channel OFETs. (**a**) Transfer characteristics of the all-solution-processed p-channel OFETs with and without SAM treatment. Output characteristic of the device (**b**) without SAM treatment and (**c**) with PFBT treatment. Output characteristics were obtained in the region of gate-to-source voltage from 0 V to −80 V with a −10 V step.

After confirming that the density of dominant carriers (i.e., electrons) could be controlled through modification the injection properties, we investigated the modulation of non-dominant carriers (i.e., holes) by the SAM treatment. The injection barrier for holes is determined by the difference between the work function of the metal and the HOMO level of the organic semiconductors. Without the SAM treatment, the hole injection barrier estimated by the Mott-Schottky model was 0.69 eV. The injection barrier for holes was reduced to 0.36 eV and increased to 0.94 eV with PFBT and TP treatment, respectively. Figure 4(a) shows the p-channel transfer characteristics of the all-solution-processed OFETs based on the electron-transport-dominant polymer, P(NDI2OD-T2). Similar to the n-channel transfer characteristics, the device performance varies considerably with the SAM treatment. With PFBT treatment, the drain-to-source on-current was increased by two-fold, and the hole mobility was also improved from 1.1 × 10^−3^ cm^2^/V·s to 1.7 × 10^−3^ cm^2^/V·s. On the other hands, the hole mobility was reduced to 9.3 × 10^−4^ cm^2^/V·s with TP treatment. The turn-on voltage of the device without SAM treatment is −30.7 V and shifts to −70.3 V and −12 V with TP and PFBT treatments, respectively, and transistor parameters in the p-channel are summarized in Table 1. The positive shift of the turn-on voltage of the device by the PFBT treatment implies an easier induction of the conducting channel, which is attributed to a higher hole density. On the basis of the threshold voltage change by the PFBT treatment (from −58.1 V to −52.7 V), the additional hole density (Δ*n*_h_) modulated by the PFBT treatment is calculated to be 1.0 × 10^11^ cm^−2^, as given by Δ*n*_h_ = *C*_GI_Δ*V*_TH_/*e*. With the TP treatment, the threshold voltage changes to −78.3 V because of the reduction of the hole density (Δ*n*_h_) by 3.78 × 10^11^ cm^−2^. Figure 4(b,c) show the output characteristics of the devices without the SAM treatment and with the PFBT treatment, respectively. As evident in Fig. 4(b), the device without the SAM treatment suffers from ambipolar behaviour and exhibits a poor injection property, which is mainly attributed to the low hole density. As a result of modulation of the hole density with the PFBT treatment, the device exhibits Ohmic properties with suppressed ambipolar behaviour.

**Figure 5:**
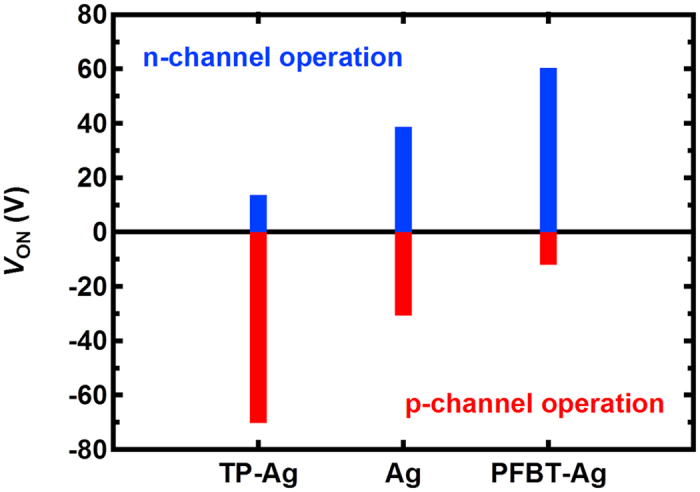
Turn-on voltage variation of the all-solution-processed OFETs according to SAM treatment in the n-channel and p-channel region.

Finally, we revisited the control of the injection-modulated charge density with SAM treatment in terms of polarity conversion in the OFETs. As previously discussed, polarity conversion is key for the fabrication of organic CMOS-like integrated circuits with a single organic semiconductor. Figure 5 shows the turn-on voltage (*V*_ON_) of the all-solution-processed OFETs with the SAM treatment in both the n-channel and p-channel regions. The polarity of the OFETs can be described by the turn-on voltages in the n-channel and p-channel region because the turn-on voltages are strongly related to the charge carrier densities. Without the SAM treatment, the device exhibits similar turn-on voltages in the n-channel and p-channel regions, implying ambipolar characteristics. Because the TP treatment induces more electrons, the absolute value of the turn-on voltage of the device is significantly reduced in the n-channel region but is increased in the p-channel region. Easier generation of the n-channel (i.e., smaller turn-on voltage) reflects the superior n-type unipolar characteristic of the device. Similarly, the modulation of the injection property by the PFBT treatment leads to a higher hole density. As a result, the absolute value of the turn-on voltage is decreased in the p-channel region and is increased in the n-channel region, indicating superior p-type characteristics of the device. On the basis of the changes in the turn-on voltages, we confirmed the feasibility of polarity conversion of the OFETs by modulation of the charge carrier densities with the SAM treatment. Because the channel material used in this work exhibits considerably mismatched electron and hole mobilities, an organic CMOS-like circuit is not demonstrated here; we performed Pspice simulation for CMOS-like inverter by using ORCAD Pspice, and found out the optimal inverter characteristic was observed when the device demension (W/L) of p-channel OFETs is 50 times larger than the one of n-channel OFETs (Fig. S1 and S2), which is too large to realize genuine CMOS-like inverter. However, the broad range of the charge density controllability by the SAM treatment suggests that an organic CMOS-like circuit can likely be achieved using this scheme if we employ a channel material with relatively well-matched electron and hole mobilities.

## Discussion

In summary, we have demonstrated control of charge carrier densities in all-solution-processed OFETs by modulating their injection property. The injection barrier was controlled using two types of SAMs, TP and PFBT, and the modified work function of the inkjet-printed Ag electrodes was confirmed by UPS measurements. The effect of injection-modulated charge carrier densities on the electrical performance of the all-solution-processed OFETs was investigated in terms of the turn-on voltage (*V*_ON_), electron field-effect mobility (*μ*_FET_), additional charge carrier density (Δ*n*_e_), and contact resistance (*R*_C_). The results indicate that SAM treatment can modulate the charge carrier density by modifying the injection barrier height. In addition, we also demonstrated the feasibility of controlling the density of non-dominant carriers (holes) by adjusting the injection barrier height with SAM treatment and observed similar trends for the turn-on voltage (*V*_ON_), hole field-effect mobility (*μ*_FET_), and additional charge carrier density (Δ*n*_h_) in the p-channel region. Modification of the injection property with a SAM is observed to be an efficient method to control the charge carrier densities in OFETs; hence, it can be used to achieve polarity conversion of the OFETs. This simple and solution-process-compatible method for the modulation of the charge carrier densities can be used to realize CMOS-like integrated circuits with a single organic semiconductor and is also useful for studying the intrinsic nature of charge transport in organic semiconductors.

## Methods

### Device fabrication

Glass was used as a starting substrate after being sequentially cleaned in acetone, isopropyl alcohol, and deionized water for 15 min each according to the conventional glass cleaning process. To improve the wetting property of the Ag ink (TEC-IJ-010, InkTec) on glass substrates, hexamethyldisilazane (HMDS) (Sigma Aldrich) was used as a surface modification layer. The source and drain electrodes were formed by inkjet-printing of Ag ink using an inkjet printer (UJ 200, UNIJET) at a substrate temperature of 80 °C. The defined channel length (*L*) and channel width (*W*) were 50 μm and 1800 μm, respectively. The inkjet-printed Ag electrodes were sintered at 150 °C for 15 min, and the sheet resistance of the inkjet-printed Ag electrodes was 0.42 Ω/□. The inkjet-printed Ag electrodes were modified by SAM prior to deposition of the active layer. An n-type polymer, poly{[*N,N′*-bis(2-octyldodecyl)-naphthalene-1,4,5,8-bis(dicar-boximide)-2,6-diyl]-*alt*-5,5′-(2,2′-bithiophene)} P(NDI2OD-T2) (Polyera ActivInk N2200), was chosen as a channel material because of its high electron mobility and good air stability^25^,^30^. P(NDI2OD-T2) was dissolved in 1,2-dichlorobenzene to a concentration of 15 mg/mL and then spin coated onto the SAM-treated Ag electrodes in an Ar-filled glove box. The P(NDI2OD-T2) films were annealed at 110 °C for 12 h in a vacuum oven, as reported elsewhere^25^. Polymethyl methacrylate (PMMA) was chosen as the gate dielectric owing to its favourable combination with P(NDI2OD-T2) and the wetting property of the Ag ink. PMMA was dissolved in propyl acetate to a concentration of 60 mg/mL and was spin coated to a thickness of 400 nm. PMMA was then annealed at 110 °C for 15 min in a N_2_ oven. As a top-gate electrode, the Ag ink was inkjet-printed onto the gate dielectric and sintered at 110 °C for 30 min.

### SAM-treatment

SAM materials were purchased from Sigma Aldrich. The inkjet-printed Ag electrodes were immersed in a toluene solution containing 10 mM of the SAM (either TP or PFBT) for 15 min. The SAM-treated samples were carefully washed with toluene to remove the physisorbed thiol molecules and then stored in a different vacuum chamber separately for 30 min to prevent cross contamination.

### Electrical characterizations

All electrical measurements were performed in an N_2_-filled glove box using a semiconductor parameter analyser (Agilent 4155 C).

## Additional Information

**How to cite this article:** Roh, J. *et al*. Injection-modulated polarity conversion by charge carrier density control via a self-assembled monolayer for all-solution-processed organic field-effect transistors. *Sci. Rep.* **7**, 46365; doi: 10.1038/srep46365 (2017).

**Publisher's note:** Springer Nature remains neutral with regard to jurisdictional claims in published maps and institutional affiliations.

## Supplementary Material

Supplementary Information

## Figures and Tables

**Table 1:** Summary of the electrical parameters of the devices with and without SAM-treatment.

Polarity	SAM-treatment	Mobility (cm^2^/V·s)	Threshold voltage (V)	Turn-on voltage (V)
n-channel	Pristine	0.021	53.7	38.8
	TP	0.11	17.3	13.6
	PFBT	2.4 × 10^−4^	60.2	60.4
p-channel	Pristine	1.1 × 10^−3^	−58.1	−30.7
	TP	9.3 × 10^−4^	−78.3	−70.3
	PFBT	1.7 × 10^−3^	−52.7	−12.0
