# Supplementary material for: Injection-modulated polarity conversion by charge carrier density control via a self-assembled monolayer for all-solution-processed organic field-effect transistors
Source: Sci Rep. 2017 Apr 12;7:46365. doi: 10.1038/srep46365 (PMC5389343; doi:10.1038/srep46365)
Supplement: Supplementary Information [file srep46365-s1.pdf]

Supplementary information for

Injection-modulated polarity conversion by charge carrier density control via a self-assembled monolayer for all-solution-processed organic field-effect transistors

Jeongkyun Roh[1], Taesoo Lee[1], Chan-mo Kang[2], Jeonghun Kwak[3], Philippe Lang[4], Gilles Horowitz[5], Hyeok Kim[6,*], Changhee Lee[1,*]

[1]Department of Electrical and Computer Engineering, Inter-University Semiconductor Research Center, Seoul National University, 1 Gwanak-ro, Gwanak-gu, Seoul 08826, Korea

[2]IT Convergence Technology Research Laboratory, Electronics and Telecommunications Research Institute, Deajeon, Korea

[3]Department of Electrical and Computer Engineering, University of Seoul, 163 Seoulsiripdaero, Dongdaemun-gu, Seoul 02504, Korea

[4]ITODYS, CNRS UMR 7086, Université Paris Diderot (Paris7), 15 rue Jean-Antoine de Baïf, 75205 Paris Cedex 13, France

[5]LPICM, Ecole Polytechnique, CNRS, 91128 Palaiseau, France

[6]Construction Equipment Technology Center, Korea Institute of Industrial Technology (KITECH), Hayang-ro 13-13, Gyeongsan 38430, Korea

Correspondence and requests for materials should be addressed to H. K. (email: fomalhout@gmail.com) or C. L. (email: chlee7@snu.ac.kr)

PSpice simulation of CMOS-like inverter

To verify the feasibility of CMOS-like inverter composed of NMOS with TP-treatment and PMOS with PFBT-treatment, we performed PSpice simulation by using ORCAD PSpice (Cadence Design Systems, Inc). ORCAD PSpice is basically a SPICE circuit simulator for simulation and verification of analog and mixed-signal circuits (PSpice is an abbreviation of Personal Simulation Program with Integrated Circuit Emphasis).

The basic parameters for the simulation such as mobilities, threshold voltages were used as the values from the Table. 1, and the capacitance per unit area of gate insulator (C_{GI}) was 3.0 nF/cm^2. The channel length modulation parameter (λ) was assumed to be 0 because the devices have relatively large channel length (50 μm). We varied the W/L ratio of NMOS and PMOS from 1:1 to 1:200 while the supply voltage was set to 80 V.

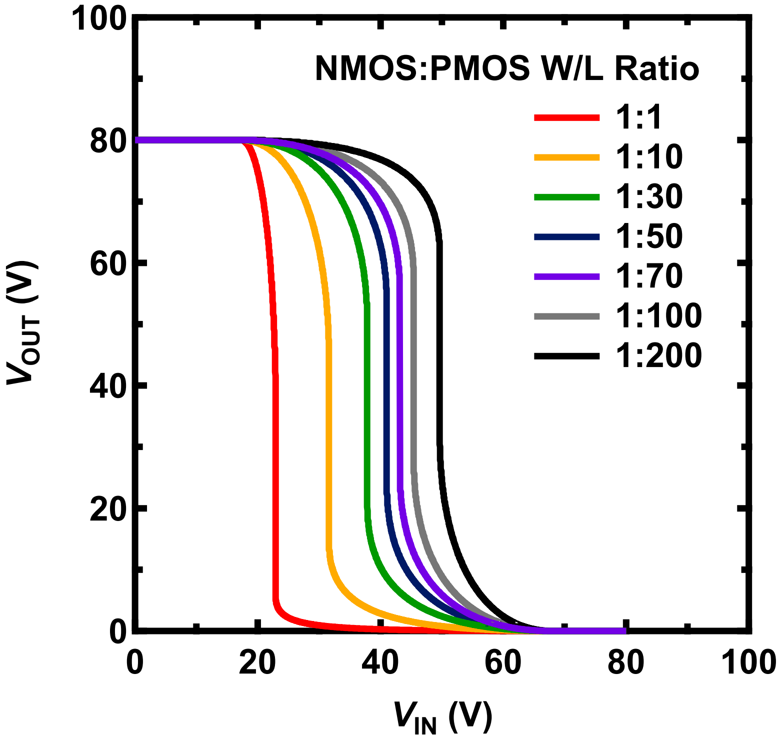

Figure S1. Simulated voltage transfer curve (VTC) of CMOS-like inverter with different width-to-length ratio of NMOS and PMOS. Simulation was performed by using the ORCAD PSpice.

Because the mobility of p-channel OFETs (PMOS) and n-channel OFETs (NMOS) are largely unbalanced, the inverter shows undesired voltage transfer behavior when they have similar device geometry; the electron mobility of the n-channel OFETs with TP-treatment was 0.11 $cm^2/V\cdot s$, however, the hole mobility of the p-channel OFETs with PFBT-treatment was only 1.7×10^{-3} $cm^2/V\cdot s$. When the *W*/*L* of PMOS is 50 times larger than the one of NMOS, the inverter shows the optimized performance. Here, we could suggest optimized design rule for PMOS and NMOS with largely different mobility. This design guide will offer a general rule to fabricate the CMOS circuit in inverter with even highly unbalanced mobilities for PMOS and NMOS, respectively.

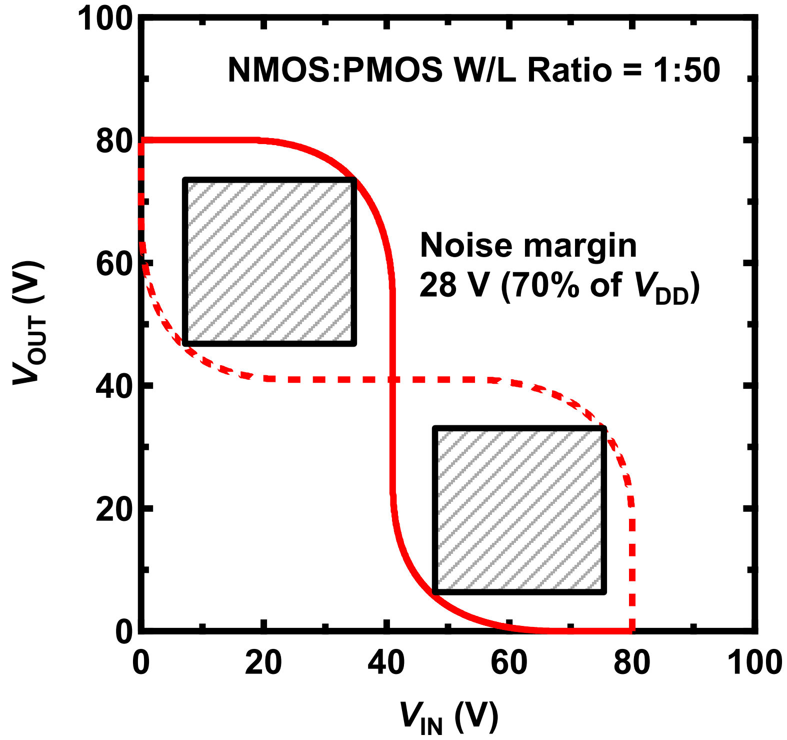

Figure S2. Optimized VTC of CMOS-like inverter obtained by PSpice simulation.

As shown in Figure S2, the CMOS-like inverter with the 1:50 W/L ratio of PMOS and NMOS shows good voltage transition characteristics with large noise margin (~70% of 1/2 V_{DD}). In particular, the large noise margin is produced with optimized design through PSpice circuit simulation. These results are of high interest because the simulation exhibits that a relatively high noise margin could be obtained even in the cased of largely unbalanced mobilities (which show up to 65 times difference) in PMOS and NMOS.
